# Supplementary material for: Structural insights into the activation of the chicken ROS1 receptor by the NEL/NICOL ligand complex
Source: Nat Commun. 2026 Feb 24;17:3124. doi: 10.1038/s41467-026-69942-8 (PMC13044245; doi:10.1038/s41467-026-69942-8)
Supplement: Supplementary file 1 — Supplementary Information [file 41467_2026_69942_MOESM1_ESM.pdf]

Supplementary Information

Structural Insights into the Activation of the Chicken ROS1 Receptor by the NEL/NICOL Ligand Complex

Weidong An[1], Xuewu Zhang[1,3,#], & Xiao-chen Bai[1,2,#]

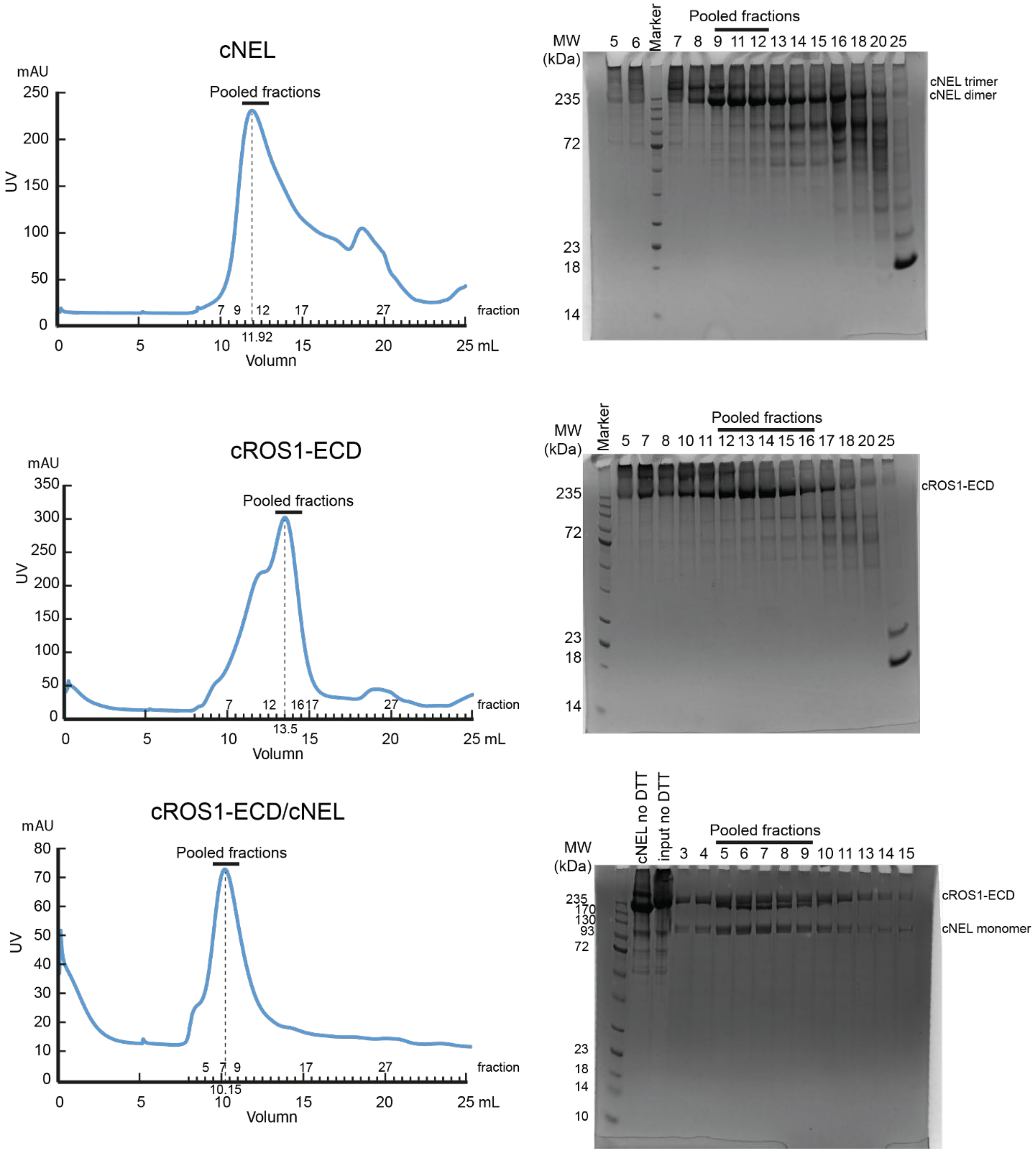

Supplementary Figure 1. Purifications of cNEL and cROS1 and reconstitution cROS1/cNEL complex. The representative SEC profiles and SDS-PAGE analyses of the cNEL, cROS1-ECD and cROS1-ECD/cNEL complex. cROS1 and cNEL were co-eluted in SEC, suggesting that the complexes were stably formed and remained intact during SEC. The SDS-PAGE of cNEL alone sample was run in the absence of DTT, while the cROS1-ECD/cNEL sample was run in the presence of DTT.

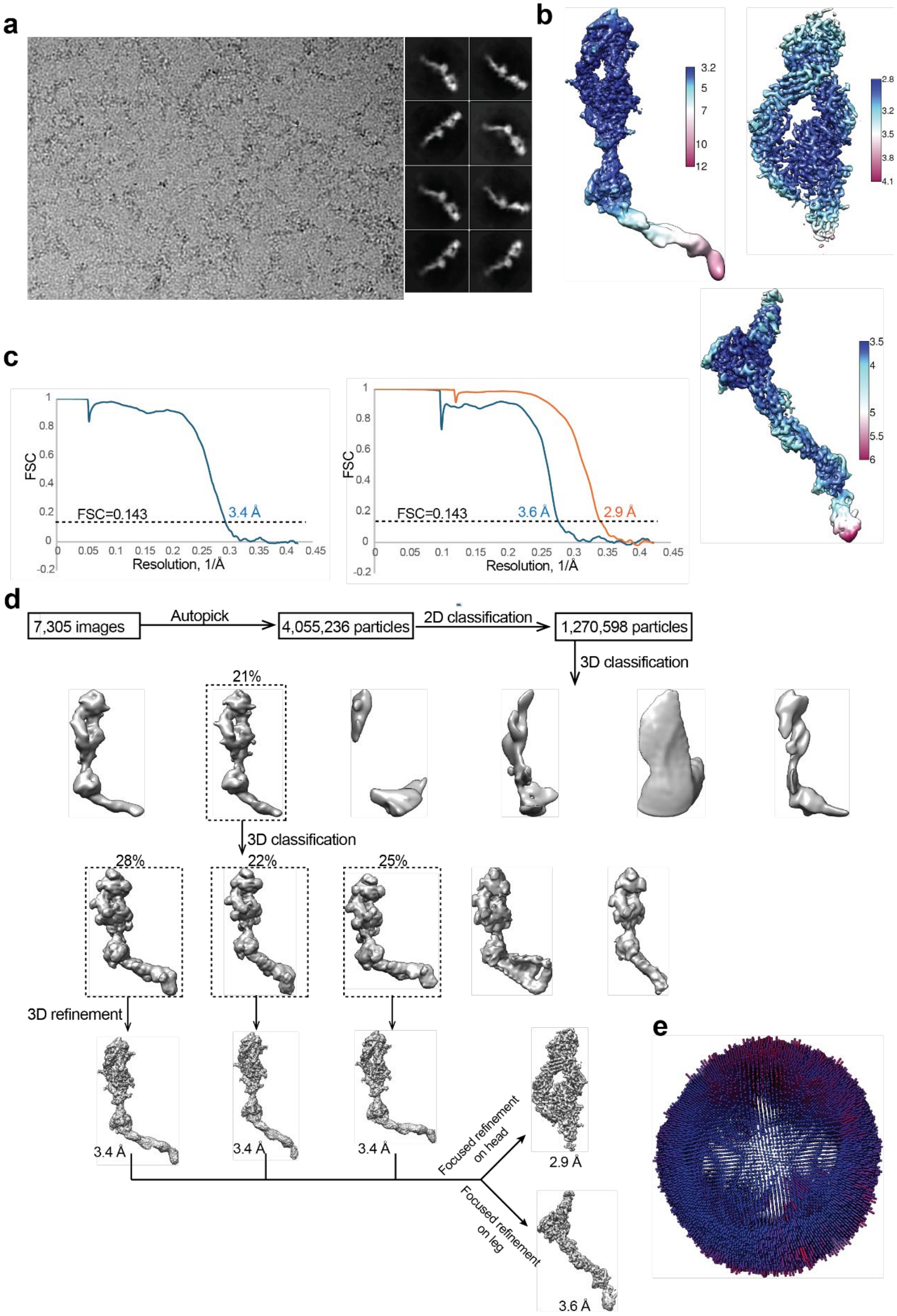

Supplementary Figure 2. Flowchart of cryo-EM data processing of cROS1 dataset.

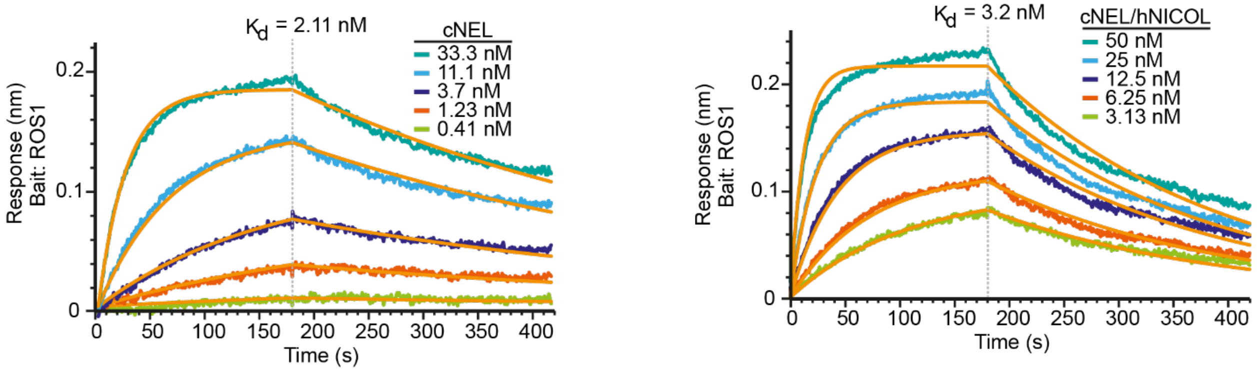

Supplementary Figure 3. Quantitative analysis of the cROS1–cNEL and cROS1–cNEL/hNICOL interactions by BLI. Binding of cNEL (Left) and cNEL/hNICOL (right) at indicated concentrations to biotinylated ROS1 coupled to BLI sensors was measured. K_d values were calculated by non-linear fitting of the data using a 1:1 binding model.

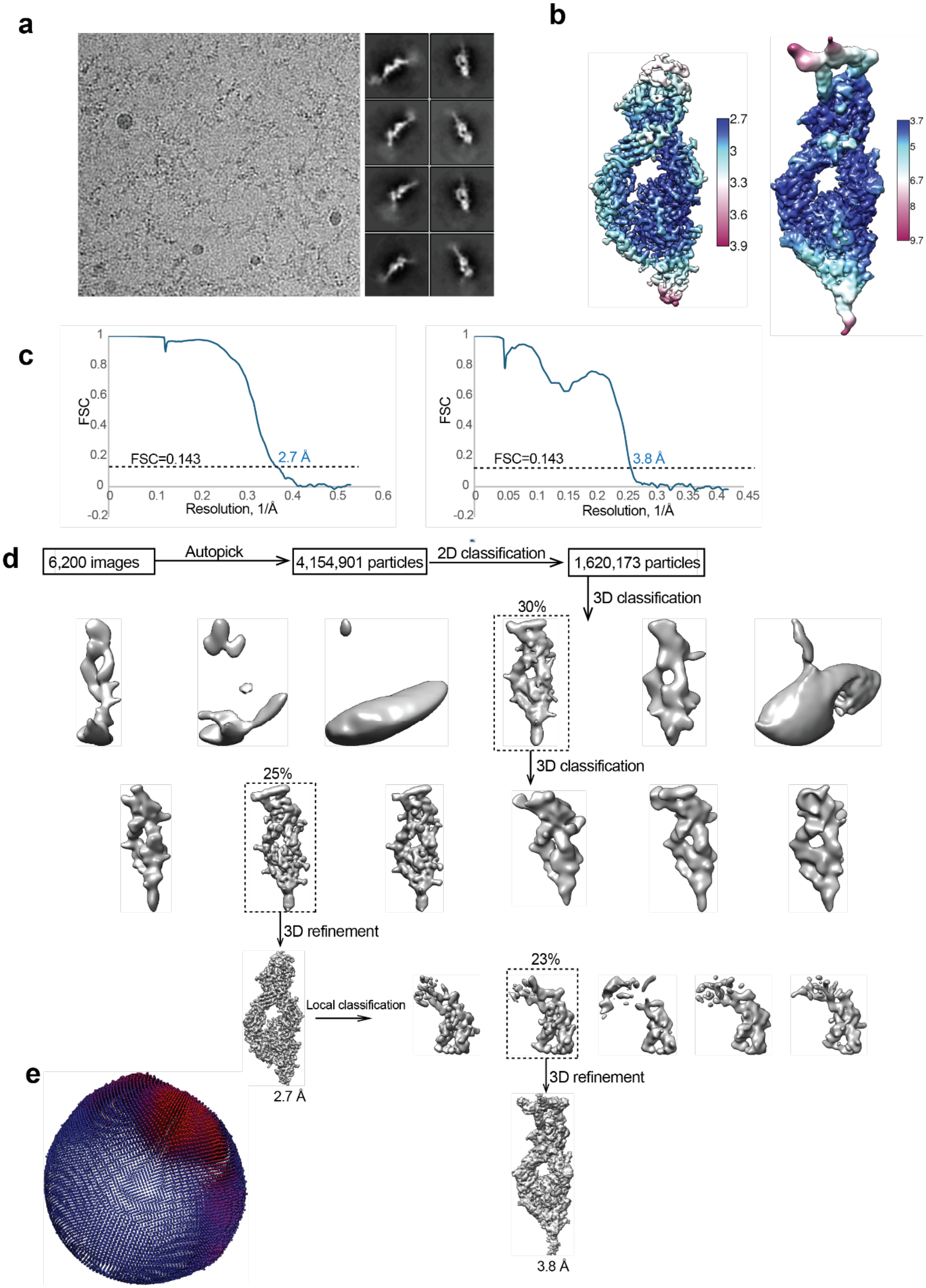

Supplementary Figure 4. Flowchart of cryo-EM data processing of cROS1/cNEL dataset.

NELL2

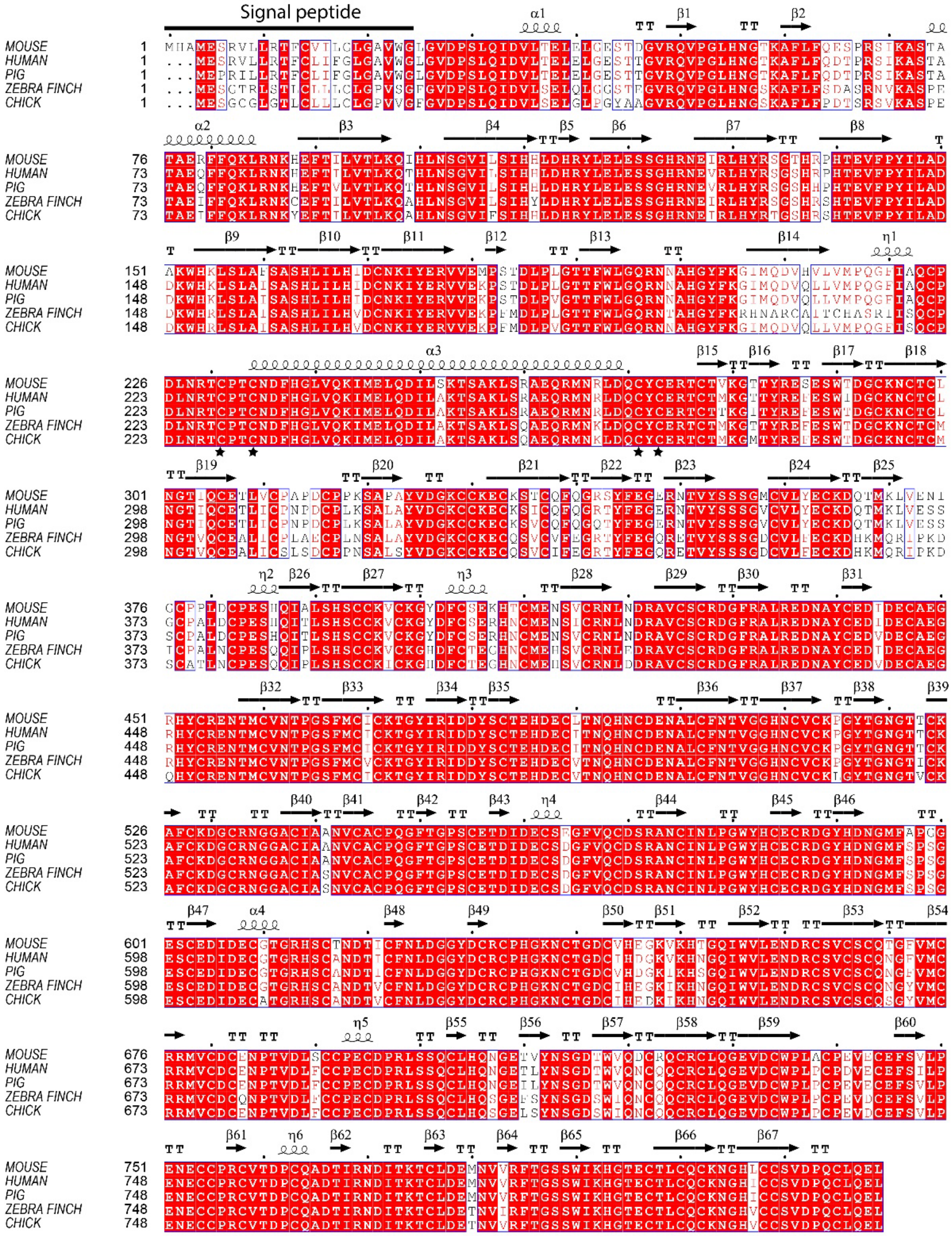
Signal peptide
MOUSE
HUMAN
PIG
ZEBRA FINCH
CHICK

NICOL

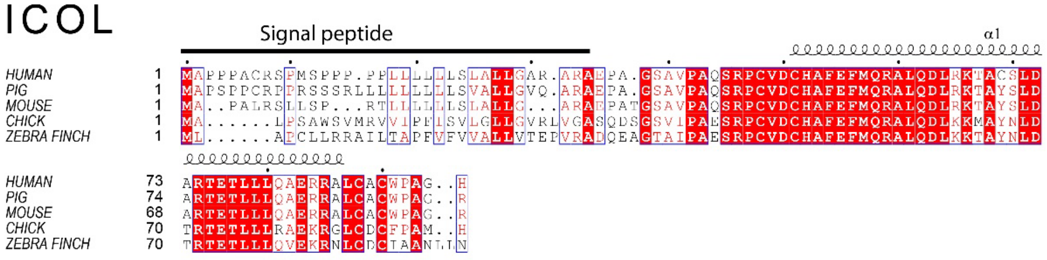
Signal peptide
HUMAN
PIG
MOUSE
CHICK
ZEBRA FINCH

Supplementary Figure 5. (**a**) A multiple sequence alignment of NELL2 from human (UniProt ID: Q99435), mouse (Q61220), pig (A0A4X1TU26), zebra finch (H0Z6H1), and chicken NEL (Q90827) was performed using Clustal Omega. Secondary structural elements from cNEL determined in this study are labeled above the sequences. The alignment was visualized with ESPript 3.0 and further edited in Adobe Illustrator. Signal peptide regions are indicated above the sequences. 4 key cystine residues that are critical for intermolecular disulfide bond formation are marked with black stars. (**b**) A multiple sequence alignment of NICOL from human (UniProt ID: Q5BLP8), mouse (Q3UR78), pig (A0A4X1T0K5), zebra finch (B5G2A1), and chicken (E1BRC3) were performed using MAFFT. Secondary structural elements from hNICOL determined in this study are labeled above the sequences. The alignment was visualized with ESPript 3.0 and further edited in Adobe Illustrator. Signal peptide regions are indicated above the sequences.

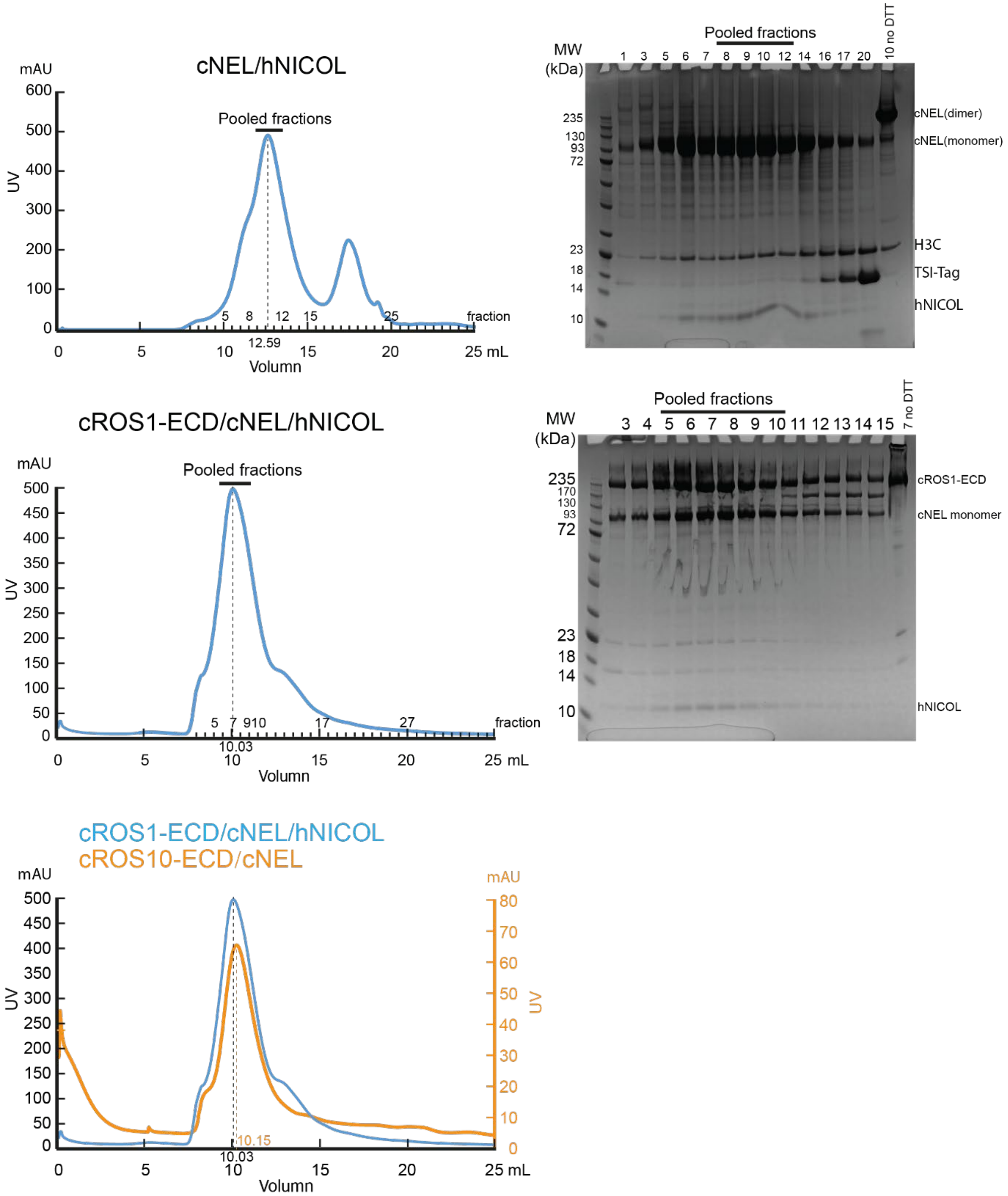

Supplementary Figure 6. Purifications of cNEL/hNICOL and reconstitution of cROS1/cNELL/hNICOL complex. The representative SEC profiles and SDS-PAGE analyses of the cNEL/hNICOL and cROS1-ECD/cNEL/hNICOL complex were showed. cROS1 and cNEL/hNICOL were co-eluted in SEC, samples were treated with DTT to resolve the NICOL band and distinguish the cROS and cNEL band. On the very right lane, a sample from a peak fraction was untreated with DTT to detect the dimeric form.

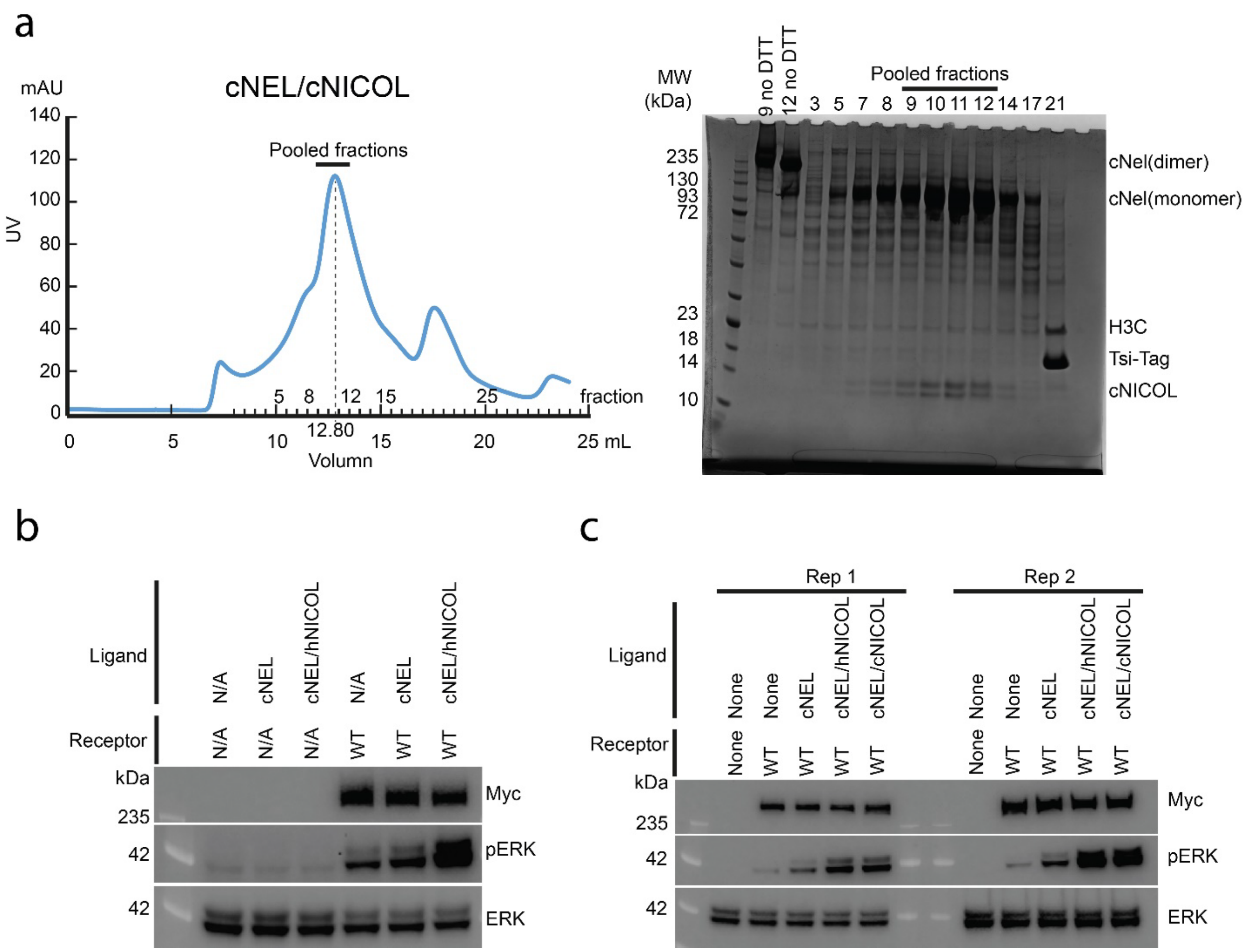

Supplementary Figure 7. (**a**) The representative SEC profiles and SDS-PAGE analyses of cNEL/cNICOL samples. Samples were treated with DTT to resolve the NICOL band. On the very left lanes, samples from a peak fraction were untreated with DTT to detect the dimeric form. (**b**) cNEL or cNEL/hNICOL complex induced ERK phosphorylation in HEK293 cells in the presence or absence of full-length Myc-tagged cROS1 expression. The results shown are representative of two biological replicates. (**c**) cNEL/cNICOL complex induced ERK phosphorylation in HEK293 cells expressing full-length myc-tagged cROS1. ERK phosphorylation levels (pERK) were assessed by western blot. Expression levels of cROS1 were monitored by anti-myc western blot. The results of two replicates are shown.

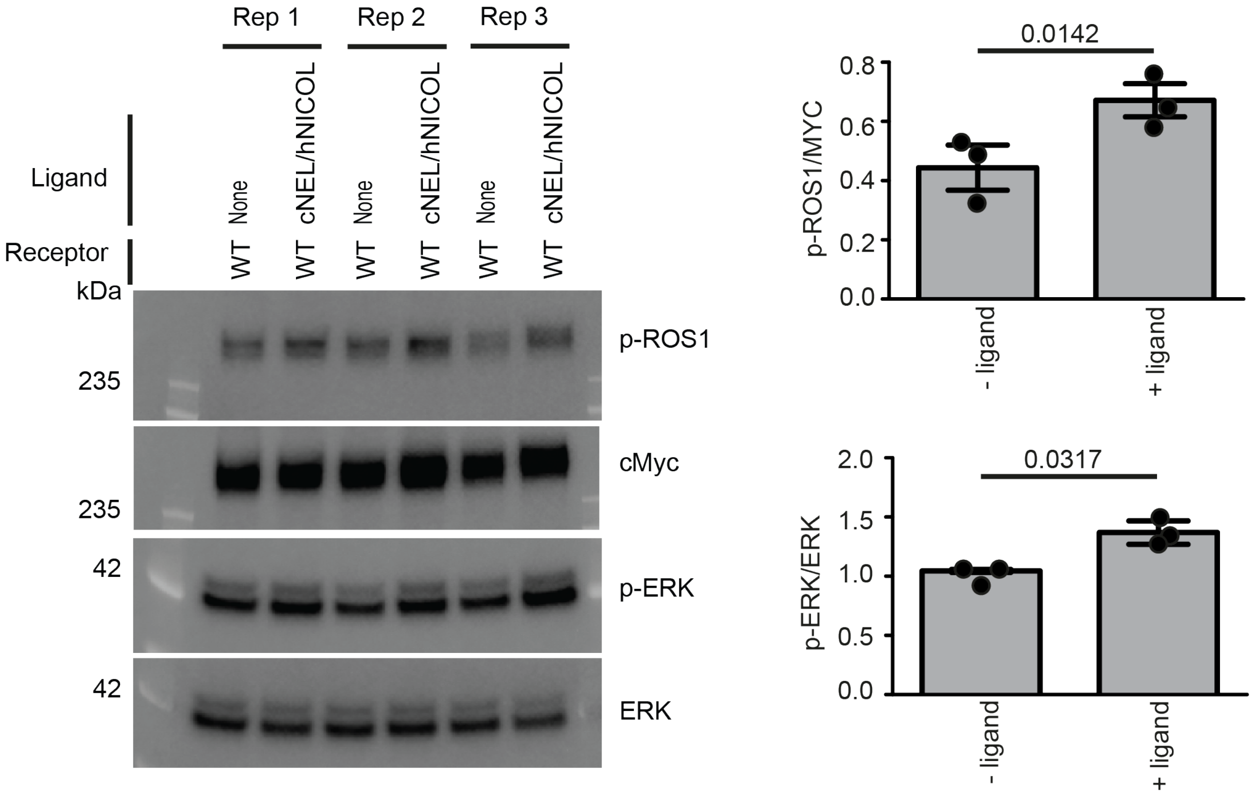

Supplementary Figure 8. cNEL/hNICOL complex induced ROS1 and ERK phosphorylation in H1299 cells expressing full-length myc-tagged cROS1. cROS1 phosphorylation levels were monitored by Phospho-ROS1 antibody. ERK phosphorylation levels (pERK) were assessed by western blot. Expression levels of cROS1 were monitored by anti-myc western blot. The phosphorylation of cROS and ERK are quantified for triplicate experiments shown in the left panel. Error bars and significance are calculated from the SD and a two-tailed t test between replicates. The results of three replicates are shown.

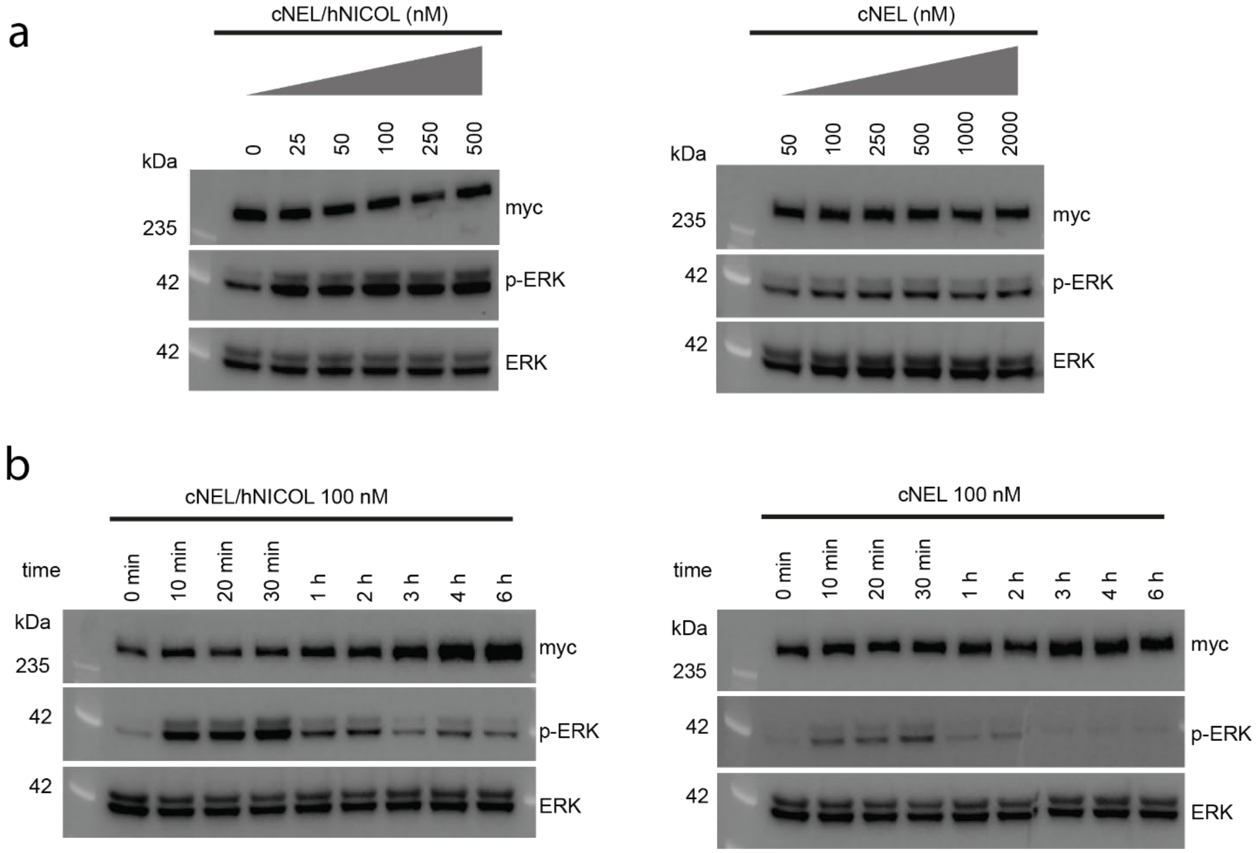

Supplementary Figure 9. (**a**) Dose-dependent activation of ERK by cNEL/hNICOL or cNEL in 293T cells expressing full-length myc-tagged cROS1. (**b**) Time course of ERK activation by cNEL/hNICOL or cNEL in 293T cells expressing full-length myc-tagged cROS1. ERK phosphorylation levels (pERK) were assessed by western blot. Expression levels of cROS1 were monitored by anti-myc western blot. The results shown in (**a**-**b**) are representative of two biological replicates.

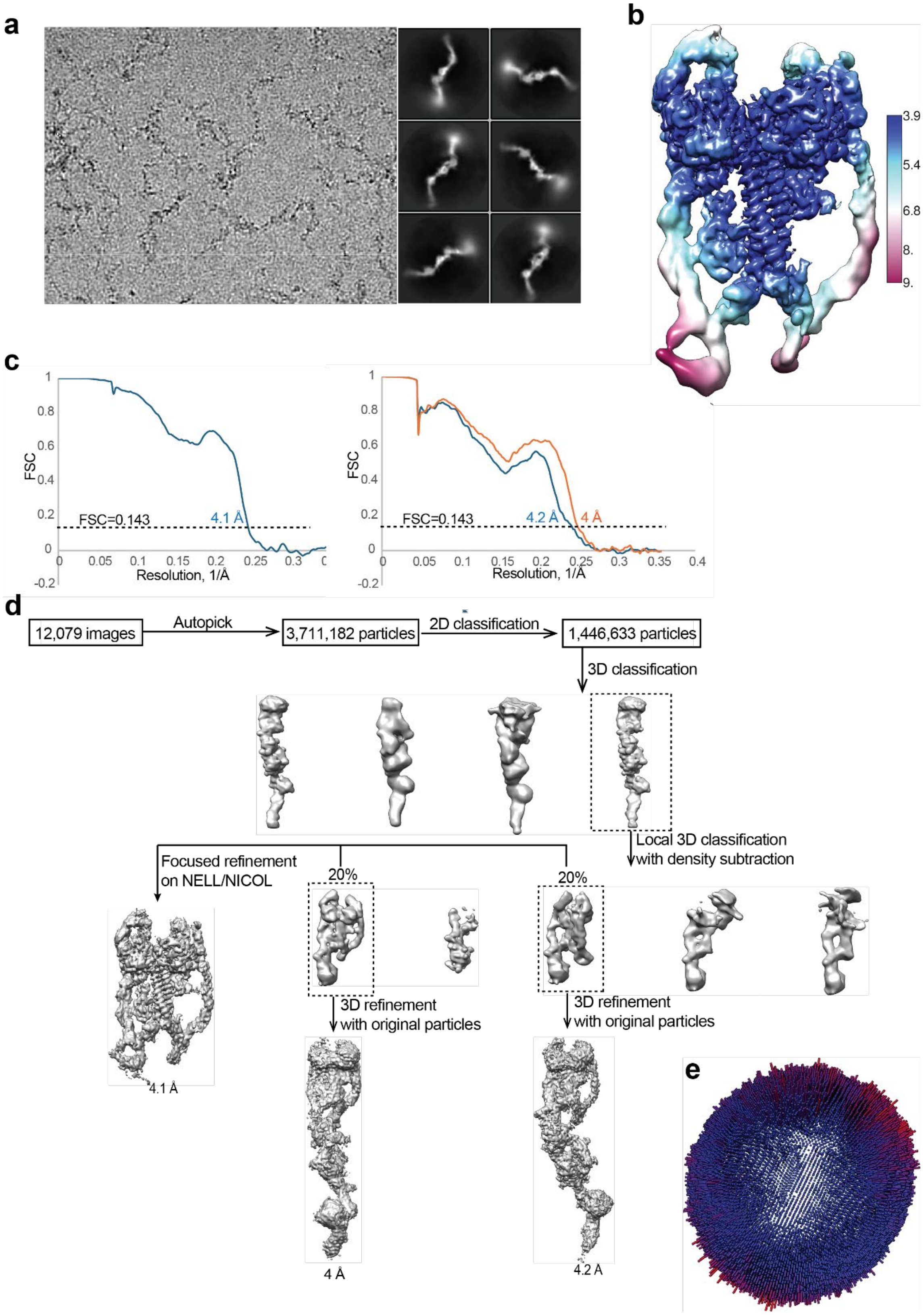

Supplementary Figure 10. Flowchart of cryo-EM data processing of cROS1/cNEL/hNICOL dataset.

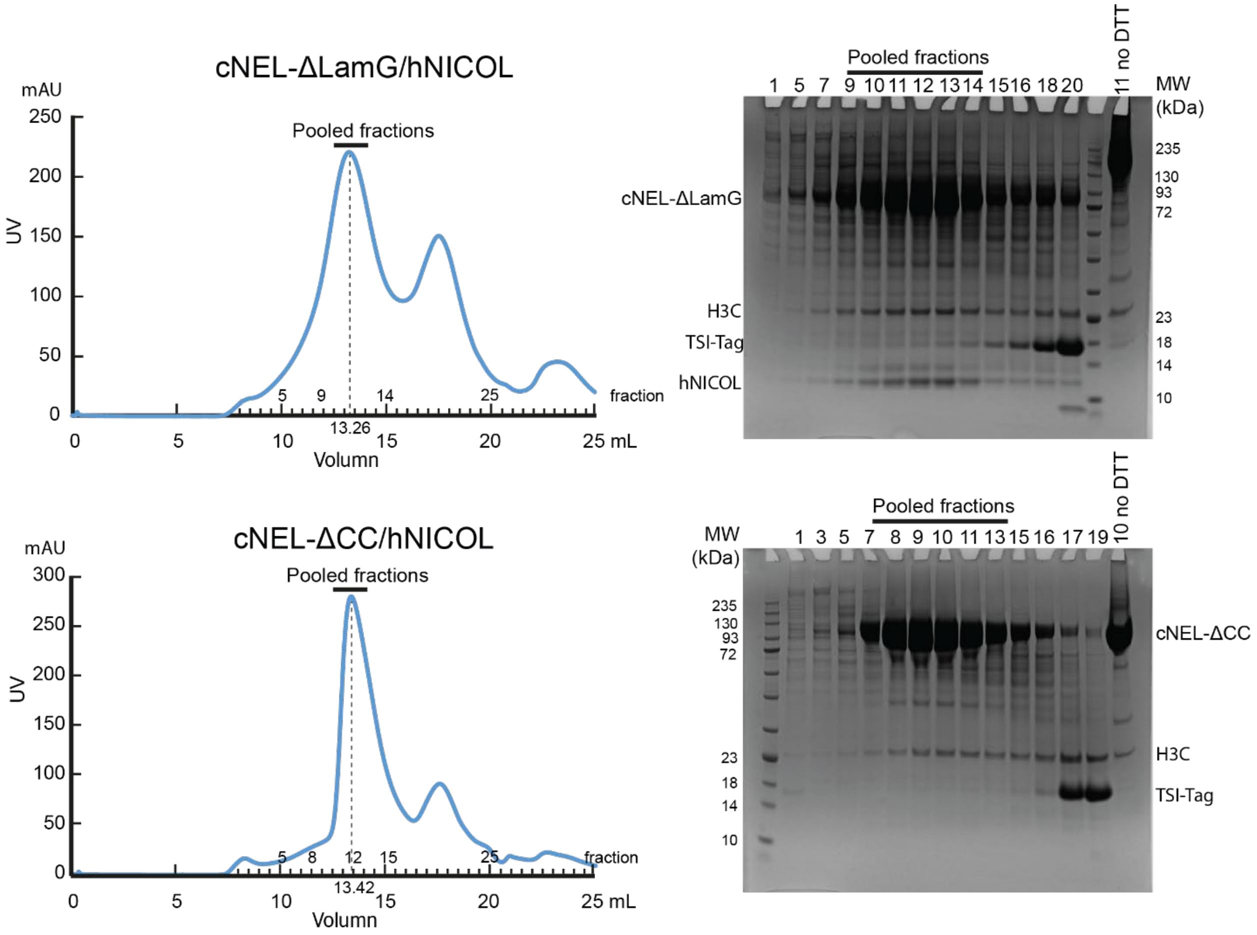

Supplementary Figure 11. Purifications of cNEL-ΔLamG/hNICOL and cNEL-ΔCC/hNICOL. The representative SEC profiles and SDS-PAGE analyses of the cNEL-ΔLamG/hNICOL and cNEL-ΔCC/hNICOL samples. Samples were treated with DTT to resolve the NICOL band. On the very right lane, a sample from a peak fraction was untreated with DTT to detect the dimeric form.

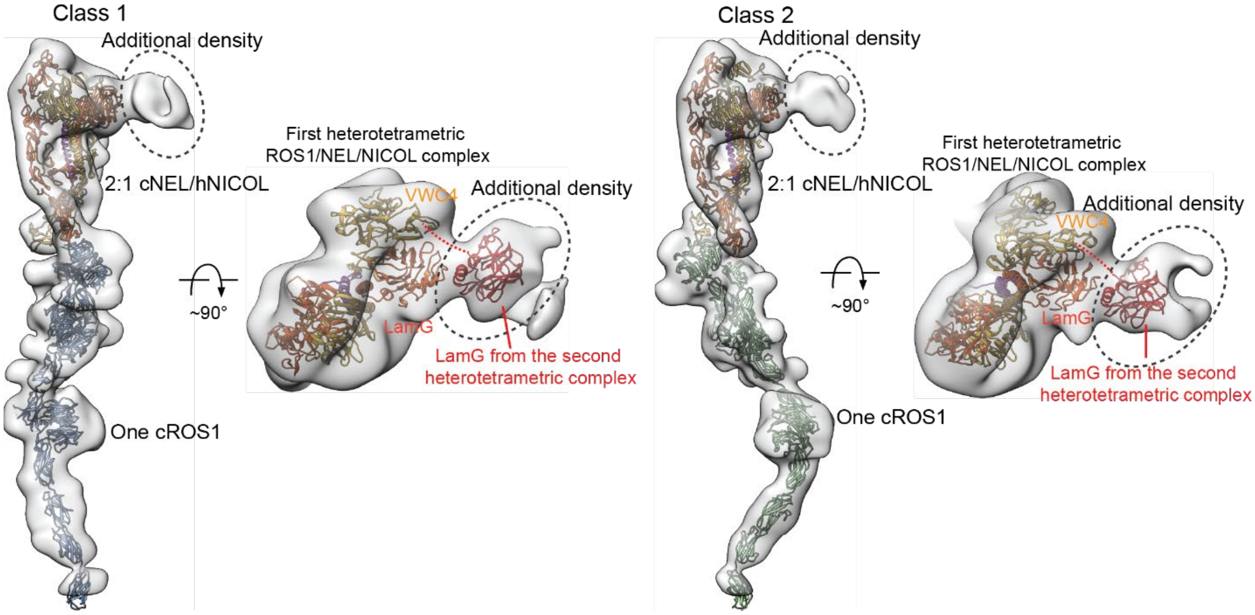

Supplementary Figure 12. The low-pass filtered cryo-EM maps of two distinct 1:2:1 cROS1/cNEL/hNICOL complexes display additional density adjacent to the VWC4 domain of cNEL protomer 2. Another LamG domain can be well fit into this additional density, suggesting that the 1:2:1 cROS1/cNEL/hNICOL complexes can further assemble into higher-order oligomers via VWC4–LamG interactions between adjacent complexes.

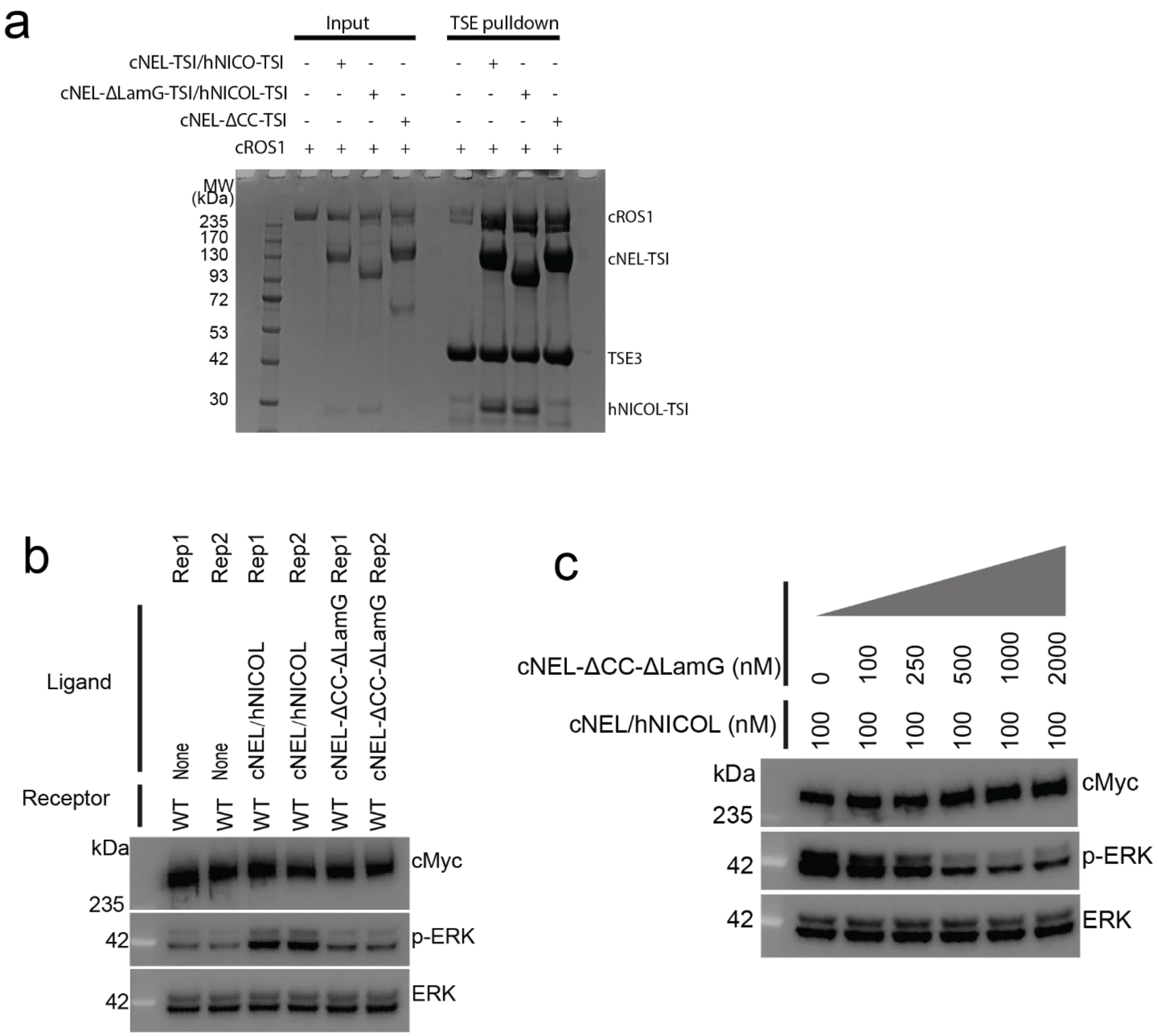

Supplementary Figure 13. (**a**) SDS-PAGE analysis of the TSE pull-down binding assay. TSI-tagged cNEL/hNICOL and various truncation mutants were immobilized on TSE beads as bait and incubated with cROS1 as prey. The results show that deletion of the LamG or CC domains does not affect the binding of cNEL to cROS1. The results shown are representative of two biological replicates. (**b**) cNEL/hNICOL and cNEL-ΔCC-ΔLamG induced ERK phosphorylation in HEK293 cells expressing full-length myc-tagged cROS1. The results of two replicates are shown. (**c**) Titrated cNEL/hNICOL by cNEL-ΔCC-ΔLamG induced ERK phosphorylation in HEK293 cells expressing full-length myc-tagged cROS1. ERK phosphorylation levels (pERK) were assessed by western blot. Expression levels of cROS1 were monitored by anti-myc western blot. The results shown are representative of two biological replicates.
